# Application of FT-IR spectroscopy using the IR Biotyper^®^ for *Leptospira*: protocol optimization and first spectral insights

**DOI:** 10.1007/s10096-026-05507-3

**Published:** 2026-04-11

**Authors:** Amanda Maria Jesus Bertani, Elaine Santos Lima, Natalia da Cunha Nobrega, Marcelo Sansone, Juliana Alves Garcia, Daniela Leite, Gisele Oliveira de Souza, Tamires Santos de Arruda, Ricardo Polatto, Eliete Calo Romero, Tania Sueli de Andrade, Karoline Rodrigues Campos, Marlon B. N. Santos, Claudio Tavares Sacchi, Marcos Bryan Heinemann, Monique Ribeiro Tiba Casas, Carlos Henrique Camargo, Roberta Morozetti Blanco

**Affiliations:** 1 Fundo Especial de Saúde para Imunização em Massa e Controle de Doenças – FESIMA, São Paulo, Brazil; 2 Instituto Adolfo Lutz, Av. Dr. Arnaldo, 351, São Paulo, SP CEP: 01246-902 Brazil; 3 Faculdade de Medicina Veterinária e Zootecnia, Universidade de São Paulo, São Paulo, Brazil

**Keywords:** *Leptospira*, FT-IR spectroscopy, IR Biotyper, Serovar discrimination, Serogroup typing, Epidemiological surveillance

## Abstract

**Purpose:**

This study aimed to optimize a Fourier Transform Infrared (FT-IR) based protocol using the IR Biotyper^®^ system for *Leptospira* strains and to provide the first spectral insights supporting its feasibility for serogroup and serovar differentiation.

**Methods:**

Reference *Leptospira* strains were cultured in Ellinghausen–McCullough–Johnson–Harris medium. A sample preparation workflow was optimized to accommodate liquid cultures, including chemical and thermal inactivation protocols to ensure biosafety. FT-IR spectra were acquired using the IR Biotyper^®^ spectrometer and analyzed by Principal Component Analysis (PCA) in the 1300–800 cm⁻¹ spectral region. Whole Genome Sequencing (WGS) was used as the reference approach for taxonomic confirmation. Identification and serovar differentiation from genomic data were performed using the jSpecies, KmerFinder, and Pathogenwatch platforms.

**Results:**

The sample preparation methods tested produced spectra suitable for analysis while ensuring biosafety. Spectral acquisition showed good reproducibility for most serovars across technical and biological replicates. Analysis of WGS performed using the jSpecies platform successfully differentiated the two major human-pathogenic serovars, Icterohaemorrhagiae and Copenhageni, whereas other bioinformatics tools showed limitations. PCA of FT-IR spectra generated by the IR Biotyper^®^ demonstrated promising discriminatory potential among the serovars and serogroups evaluated, including closely related serovars such as Icterohaemorrhagiae and Copenhageni and serovars within the Australis and Sejroe serogroups.

**Conclusion:**

The IR Biotyper offers a rapid FT-IR approach for *Leptospira* typing. Under the experimental conditions evaluated, the method allowed differentiation of some serovars. Although preliminary, these findings suggest that FT-IR may represent a promising complementary tool for epidemiological surveillance of leptospirosis.

**Supplementary Information:**

The online version contains supplementary material available at 10.1007/s10096-026-05507-3.

## Introduction

 Leptospirosis is a globally distributed zoonotic disease caused by spirochetes of the genus *Leptospira* and represents a major public health concern within the One Health framework. Its relevance stems from the wide range of susceptible hosts, including humans, domestic animals, and wildlife, and its strong association with environmental factors such as flooding, heavy rainfall, and inadequate sanitation. The clinical spectrum is highly heterogeneous, and is closely linked to the diversity of circulating serovars, reinforcing the need for integrated surveillance and control strategies [1, 2].

Transmission occurs primarily through direct contact with infected animals or exposure to water and soil contaminated with pathogenic leptospires, particularly during flooding events [3–5]. Rodents act as the main reservoirs, shedding these bacteria into the environment while remaining asymptomatic [6].

*Leptospira* serovars are often associated with specific animal reservoirs; therefore, knowledge of circulating serovars is essential for effective surveillance, control, and monitoring of leptospirosis [1, 7]. This information is also critical for vaccine development and optimization, as currently available animal vaccines do not provide cross-protection between serovars and no human vaccine is available in Brazil [8–10]. In addition, accurate knowledge of locally prevalent serovars underpins laboratory diagnosis, particularly the microscopic agglutination test (MAT), which remains the reference method for leptospirosis [11].

The genus *Leptospira* currently comprises 78 genomically distinct species and exhibits marked antigenic diversity, with more than 300 serovars grouped into serogroups [12]. Serovar classification is based on structural variation in the lipopolysaccharide (LPS) and relies on serological relatedness determined by the cross-agglutinin absorption test (CAAT) [13–15]. Among pathogenic species, *Leptospira interrogans* is the most frequently reported worldwide, with serovars Icterohaemorrhagiae and Copenhageni, both belonging to the Icterohaemorrhagiae serogroup, being consistently associated with severe disease in humans and animals [7, 16–18].

Although CAAT remains the gold standard for serovar identification, its routine use is limited by the need for extensive panels of reference antisera produced in rabbits, live antigen cultures, and specialized technical expertise, making it labor-intensive and costly [3]. Several molecular alternatives, such as pulsed-field gel electrophoresis (PFGE) and multilocus sequence typing (MLST), have been developed [16, 19–21]; however, these approaches fail to reliably discriminate between closely related serovars of major epidemiological relevance, particularly Icterohaemorrhagiae and Copenhageni.

Whole-genome sequencing (WGS) for *Leptospira* [22] has provided definitive resolution for these serovars, revealing distinct genomic clusters and identifying a statistically significant phase-shift mutation in the lic12008 gene associated with LPS biosynthesis in serovar Icterohaemorrhagiae. Despite its unparalleled discriminatory power, the implementation of WGS in routine or large-scale surveillance remains constrained by cost, infrastructure requirements, and turnaround time, highlighting the need for complementary, rapid, and cost-effective phenotypic methods.

In this context, Fourier Transform Infrared (FT-IR) spectroscopy has emerged as a promising tool for microbial typing and epidemiological applications, including characterization of LPS composition in *Leptospira* serovars [14]. The method is based on spectral signatures generated by infrared absorption of chemical bonds within the sample, enabling discrimination of isolates through comparative analysis of their spectral fingerprints [23]. The IR Biotyper^®^ system (Bruker Daltonics, Bremen, Alemanha ) applies FT-IR technology within a standardized, rapid, and reproducible workflow and has demonstrated effectiveness for typing a range of bacterial pathogens, with performance comparable to established methods such as MLST and WGS [24–28].

To date, however, FT-IR using the IR Biotyper^®^ has not been applied to the characterization of *Leptospira* spp. Therefore, this study aimed to optimize an FT-IR–based protocol using the IR Biotyper^®^ system for *Leptospira* strains and to provide the first spectral insights supporting its feasibility for serogroup and serovar discrimination.

## Methods

### Study design

This retrospective study evaluated the feasibility of applying Fourier Transform Infrared (FT-IR) spectroscopy using the IR Biotyper^®^ system (Bruker) for spectral acquisition of *Leptospira* serovars. Our primary objective was to establish a standardized sample-preparation workflow tailored to *Leptospira* spp. and to generate preliminary spectral data covering the major serogroups and serovars circulating worldwide. Whole-genome sequencing (WGS) was performed to confirm the taxonomic identity of the strains included in this study, serving as a reference for the interpretation of the FT-IR spectra.

### Strains and culture conditions

This study included reference strains routinely used for the microscopic agglutination test (MAT) at the Leptospirosis Laboratory of the Instituto Adolfo Lutz (IAL) for human serological diagnosis, as well as strains maintained at the Bacterial Zoonoses Laboratory of the School of Veterinary Medicine and Animal Science, University of São Paulo (LZB-USP), for serological diagnosis in animals. A total of 22 serovars representing 17 serogroups and 4 species were incorporated into the analysis (Table 1), each corresponding to a different *Leptospira* serovar. Thus, only one representative strain per serovar was analyzed in the present study. Some serogroups, such as Sejroe, were represented by serovars from different *Leptospira* species, reflecting the known complexity of serological and genomic classification within the genus. The IAL strains originated from the *Leptospira* Collection (CLEP) of the National Reference Center for Leptospirosis at Instituto Oswaldo Cruz (IOC)-RJ, Brazil, and from the IAL’s Microorganism Collection Nucleus (NCMO). The LZB-USP strains were sourced from the laboratory’s own isolates and from internationally recognized collections, including the Centers for Disease Control and Prevention (CDC, Atlanta, USA), the Institute Pasteur (Paris, France), and the Federal Institute for Consumer Health Protection and Veterinary Medicine (BgVV, Berlin, Germany). All strains were cultivated under standardized conditions to minimize potential variability related to culture parameters. Briefly, all isolates were grown in liquid Ellinghausen–McCullough–Johnson–Harris (EMJH) medium at 30 °C +/- 2 °C, under aerobic conditions, ensuring that the same culture medium and incubation temperature were maintained throughout all experiments prior to FT-IR analysis. For biological replication, three consecutive weekly subcultures were prepared for each strain.

**Table 1:** Strains of *Leptospira* spp. tested by IR Biotyper

Species	Serogroup	Serovar	Strain	Origin
*L. interrogans*	Australis	Australis	Ballico	IAL
		Bratislava	Jez Bratislava	FMVZ-USP
	Autumnalis	Autumnalis	Akiyami A	IAL
	Bataviae	Bataviae	Swart	IAL
	Canicola	Canicola	Hond Utrecht IV	IAL
	Icterohaemorrhagiae	Copenhageni	M20	IAL
		Icterohaemorrhagiae	RGA	IAL
	Sejroe*	Hardjo	Hardjoprajitno	IAL
		Wolffi	3705	IAL
	Hebdomadis	Hebdomadis	Hebdomadis	IAL
	Pomona	Pomona	Pomona	IAL
	Pyrogenes	Pyrogenes	Salinem	IAL
	Djasiman	Djasiman	Djasiman	IAL
*L. borgpetersenii*	Javanica	Javanica	Veldrat Bataviae 46	IAL
	Sejroe*	Sejroe	M84	IAL
		Hardjobovis	Sponselee	FMVZ-USP
	Tarassovi	Tarassovi	Perepelitsin	IAL
	Ballum	Castellonis	Castellon-3	FMVZ-USP
	Celledoni	Whitcombi	Whitcombi	FMVZ-USP
*L. kirschneri*	Cynopteri	Cynopteri	3522 C	IAL
	Grippotyphosa	Grippotyphosa	Moskva V	FMVZ-USP
*L. noguchii*	Panama	Panama	CZ-214	FMVZ-USP

*The Sejroe serogroup comprises serovars from different *Leptospira* species and is therefore presented under each corresponding species in the table

### Whole-Genome Sequencing (WGS)

WGS was employed as a reference method to confirm the taxonomic identity (species and serovar) of all *Leptospira* strains included in this study, ensuring that the isolates used for FT-IR protocol optimization using IR Biotyper^®^ analysis were correctly characterized prior to downstream experiments.

#### DNA extraction

Cultures grown for seven days in liquid EMJH medium were centrifuged at 10,000 x g for 10 min, washed twice with Phosphate buffered saline (PBS) pH 7.4, and processed for genomic DNA extraction using the QIAamp^®^ DSP DNA Blood Mini kit (QIAGEN), following the manufacturer’s instructions.

Pellets were resuspended in 180 µL ATL buffer and 20 µL proteinase K, followed by incubation at 56 °C for 2 h. Subsequently, 200 µL AL buffer and 200 µL ethanol (96–100%) were added, and the mixture was vortexed and transferred to silica columns for washing and elution. DNA was eluted in 40 µL of ultrapure water.

#### DNA quality assessment

DNA concentration and purity were measured using a NanoDrop One spectrophotometer (Thermo Scientific, Waltham, MA, USA) and Qubit^®^ 3.0 fluorometer (Thermo Scientific, Waltham, MA, USA). Acceptable extracts showed 260/280 ratios > 1.8 and 260/230 ratios > 2.0. DNA integrity was confirmed using 2% E-gel^®^ agarose electrophoresis.

#### Library preparation and sequencing

Genomic libraries were prepared using the Illumina DNA Prep kit (Illumina, San Francisco, CA, USA), following the manufacturer’s instructions (Reference Guide: 1000000025416 v09). Sequencing runs were performed using the NextSeq Reagent Kit P1 (300 cycles, 2 × 151-bp paired-end) in accordance with the manufacturer’s guidelines (NextSeq System User Guide 1000000109376 v06).

#### Bioinformatic analysis

FASTQ files were evaluated for quality and GC content using FastQC (v0.12.1) and MultiQC (v1.11). Genome assembly was performed with CLC Genomics Workbench (QIAGEN), and assembly metrics were examined using QUAST (v5.2.0). Final assemblies in FASTA format were used for downstream analysis.

Strain identification and taxonomic confirmation were conducted using the jSpecies platform for average nucleotide indentity (ANI) calculations (Z-score) [29], the KmerFinder 3.2 tool curated by the Center for Genomic Epidemiology (CGE), which identifies species based on k-mer matches against curated reference genomes [30], and Pathogenwatch, developed and maintained by the Centre for Genomic Pathogen Surveillance (CGPS), which assigns species and serovars through genome-wide comparative analysis integrated with curated epidemiological databases [31]. The simultaneous use of these platforms enabled a comparative assessment of their performance in species- and serovar-level identification of the analyzed strains.

Genomes were deposited at National Center for Biotechnology Information (NCBI) under the Bioproject PRJNA1423741.

### FT-IR spectroscopy using the IR Biotyper^®^

#### Sample preparation

Sample preparation followed an adapted workflow based on published IR Biotyper^®^ protocols but modified to accommodate *Leptospira* spp., which grow primarily in liquid medium. *Leptospira* spp. grew in liquid EMJH medium and different culture ages (5, 7, and 9 days) and starting volumes (5 mL and 10 mL) were evaluated.

All de volume of culture was centrifuged at 10,000 x g for 10 min, and pellets were washed twice with ultrapure water to remove medium components that might interfere with infrared absorbance.

Pellets were processed either in microtubes supplied with the IR Biotyper^®^ kit or in standard 1.5-mL microtubes.

For chemical inactivation and sample preparation, each pellet was resuspended in 50 µL of 70% ethanol and 50 µL of ultrapure water. Heat inactivation at 50–56 °C for 30 min [32] was also evaluated as an alternative to ethanol treatment. Fifteen microliters of the bacterial suspension was spotted in five technical replicates onto the IR Biotyper target plate and either dried at 35 °C for 15 min or allowed to dry at room temperature. Spectra were acquired immediately after drying or after storage of the target plate at room temperature until the following day. Additional aliquots of IR Biotyper suspension were stored at room temperature, protected from light, and analyzed after 2, 4, and 7 days to assess spectral stability.

To confirm biosafety, aliquots of each prepared sample were reinoculated into fresh EMJH medium and incubated at 30 °C for up to 30 days. Absence of growth was taken as evidence of successful inactivation.

Samples yielding insufficient biomass for spectral acquisition (less biomass to spot) were retested using an increased spotting volume. In these cases, 20 µL of the bacterial suspension was applied onto the IR Biotyper target plate instead of 15 µL, while all other preparation and acquisition parameters remained unchanged. To prevent volumes of 20 µL from exceeding the application edges on the plate, 15 µL or 10 µL were placed first, and then, after drying began, another 5 µL or 10 µL, respectively, were added.

#### Spectral acquisition and data processing

Spectra were acquired using the IR Biotyper^®^ spectrometer (Bruker) in transmission mode over the 4000–500 cm⁻¹ range. For each strain, biological triplicates and technical quintuplicates were analyzed.

Spectral acquisition was performed in OPUS software (Bruker), and all preprocessing was conducted in the IR Biotyper^®^ Software (Bruker Daltonics). The Principal Component Analysis (PCA) score matrices were used to evaluate all the spectral. PCA analyses were performed with a variance threshold of 0.95 and a maximum of 20 principal components (PCA 0.95/20). Following manufacturer-recommended workflows and previously published IR Biotyper^®^ studies, the spectra were trimmed to the 1300–800 cm⁻¹ carbohydrate fingerprint region and vector-normalized to minimize biomass-related variability.

Instrument performance was monitored by analyzing test standards (IRTS 1 and IRTS 2 controls) before each run. Background spectra were acquired between each measurement.

During spectra acquisition the IR Biotyper performs a check of different spectral properties, e.g. absorbance intensity, signal-to-noise ratio and water vapor disturbance.

This is called the spectral quality test. The IR Biotyper^®^ classification quality test scoring system is as follows: (a) Green score: highly reliable classification; (b) Orange score: moderately reliable classification; (c) Red score: unreliable classification. Replicates that acquired a green or orange score were included in the analysis.

## Results

### Strain identification by WGS and taxonomic confirmation

All strains demonstrated satisfactory identification across the three WGS platforms employed. The jSpecies platform exhibited the highest performance in species-level identification for *Leptospira* spp., and crucially, at the serovar level. Analysis of the Average Nucleotide Identity (ANI) Z-scores revealed that the majority of values exceeded 0.999 (above the cutoff), indicating a high degree of nucleotide identity among the examined genomes. Specifically, jSpecies was successful in differentiating between the two genetically related serovars associated with major human disease: Icterohaemorrhagiae and Copenhageni. In contrast, the KmerFinder and Pathogen watch platforms primarily showed better performance at the species level, demonstrating limitations in distinguishing between these two serovars (Supplementary Table 1).

As WGS was used in the present study to confirm the taxonomic identity of the strains included in the FT-IR analysis, these results demonstrate its effectiveness in ensuring the correct characterization of the isolates prior to downstream applications.

### Spectral quality and preliminary assessments

The methodological optimization confirmed that all evaluated sample preparation approaches, including variations in culture age, starting volume, inactivation method, drying conditions, and acquisition timing, yielded spectra of adequate quality. Drying of the spotted samples either at 35 °C or at room temperature did not affect spectral quality or reproducibility. In addition, delayed acquisition performed on the day following spotting produced spectra comparable to those acquired immediately after drying, demonstrating the stability of the prepared target plates.

The IR Biotyper system consistently assigned Orange quality scores, indicating moderately reliable classification, to samples presenting homogenization or biomass-related limitations, thereby allowing corrective actions prior to large-scale data acquisition. For samples initially classified as having less biomass to spot, increasing the spotting volume from 15 µL to 20 µL resulted in a clear improvement in spectral quality, with quality scores shifting from Orange to Green, indicating high quality classification. After test all the strains with 20 µL, considering the 330 spectra generated in this study, corresponding to 15 spectra for each of the 22 strains analyzed (five technical replicates and three biological replicates), 241 (73.03%) achieved Green quality scores and 89 (26.96%) were classified as Orange. Among the spectra classified as Orange, 74 were a problem of absorbance associated with insufficient biomass; six were a problem of signal-to-noise associated with homogenization issues; and nine with a combination of both factors.

Crucially, the viability assays conducted after both chemical (70% ethanol) and thermal (50–56 °C for 30 min) inactivation protocols confirmed the complete absence of motile organisms and no culture recovery after 30 days of incubation.

Since all evaluated conditions produced comparable spectral quality, the following selected protocol was chosen based on both spectral performance and practical considerations: 7-day culture, initial volume of 5 mL, chemical inactivation, standard microtubes and 20 µL in the plate. PCA, configured with a variance threshold of 0.95 and a maximum of 20 principal components (PCA 0.95/20), was performed on the carbohydrate fingerprint region (1300–800 cm^− 1^).

Representative spectra for each serovar, as well as an overlay including all serovars, are shown in Supplementary Material (Supplementary Fig. 1 and Supplementary Fig. 2, respectively).

### Spectral discrimination of serovars belonging to the same serogroup

**Fig. 1:**
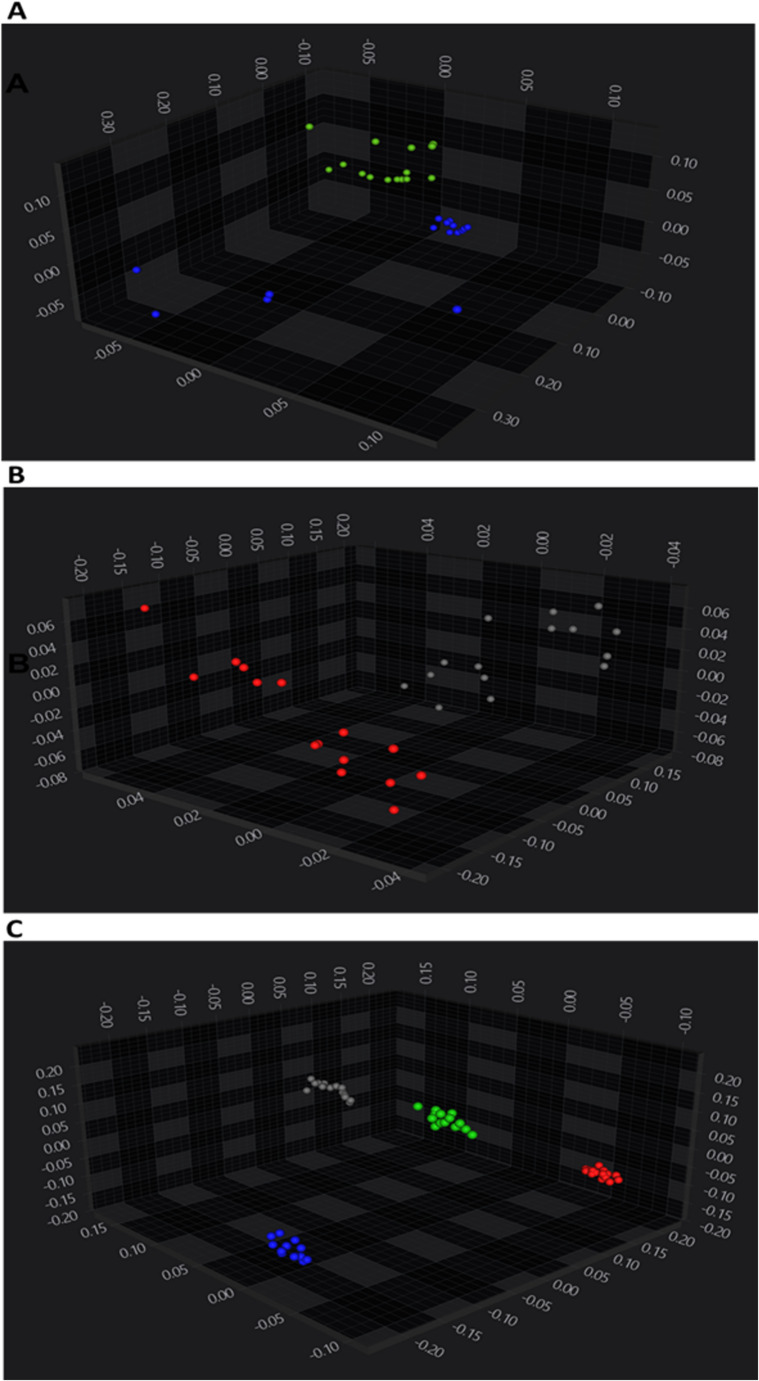
Three-dimensional PCA scatter plot representation of FT-IR results using IR Biotyper. The single circle represents one single spectrum (biological and technical replicates). **A**: Serovars Icterohaemorrhagiae in Blue and Copenhageni in Green, within Icterohaemorrhagiae serogroup; **B**: Serovars Australis in Grey and Bratislava in Red, within Australis serogroup; **C**: serovars Hardjo in Grey, Hardjobovis in Blue, Sejroe in Green, and Wolffi in Red, within Sejroe serogroup

PCA analyses of selected serovars from the Icterohaemorrhagiae (Panel A), Australis (Panel B), and Sejroe (Panel C) serogroups are shown in Fig. 1. PCA results suggested potential discrimination among serovars within their respective serogroups, including related serovars Icterohaemorrhagiae and Copenhageni (Fig. 1A), belonging to the Icterohaemorrhagiae serogroup. Furthermore, PCA also showed discrimination within the Australis serogroup (Fig. 1B), with separation between the Australis and Bratislava serovars, and within the Sejroe serogroup (Fig. 1C), separating the Hardjo, Hardjobovis, Sejroe, and Wolffi serovars.

### Analysis of all strains by serovar and serogroup

**Fig. 2:**
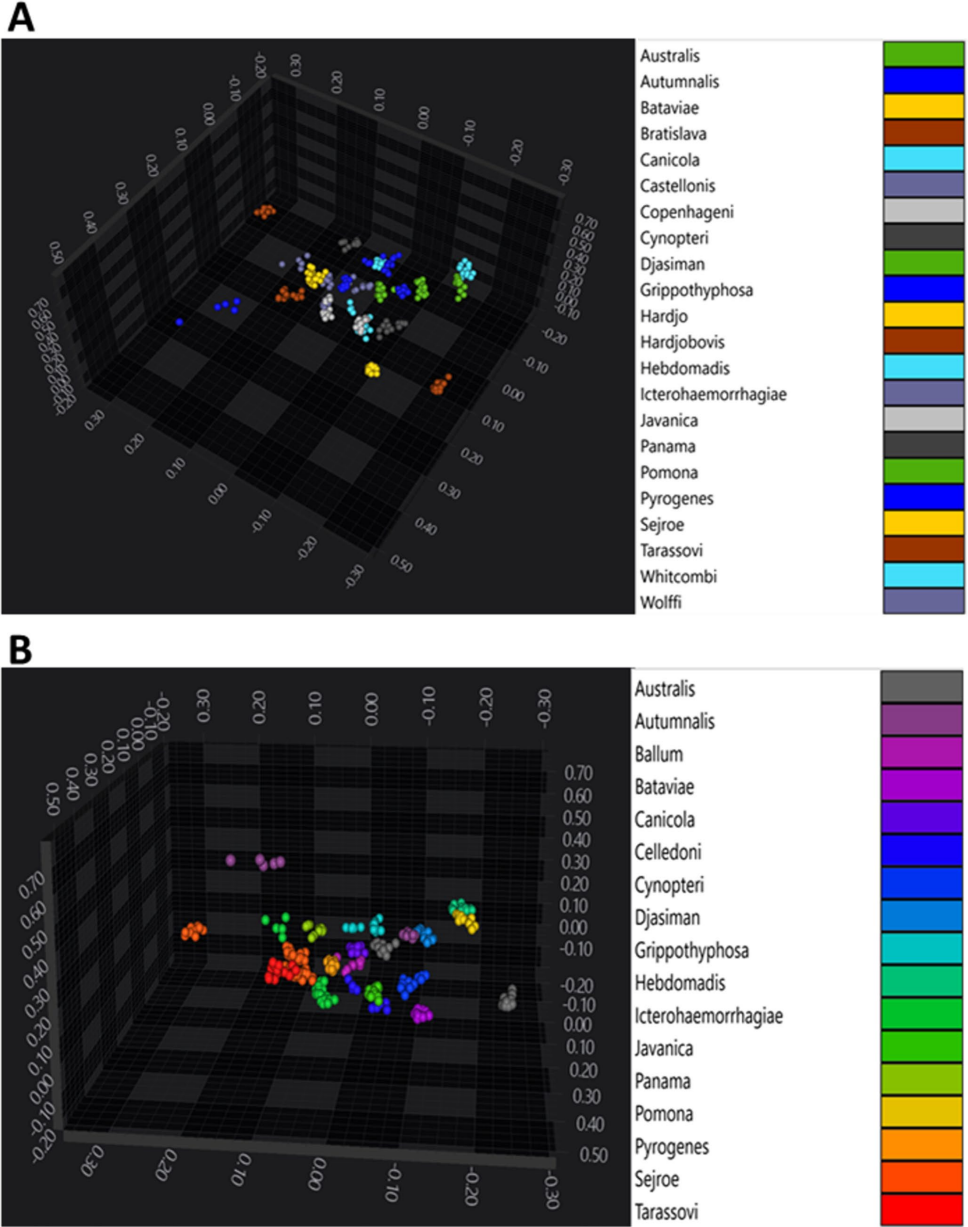
Three-dimensional PCA scatter plot representation of FT-IR results using IR Biotyper. The single circle represents one single spectrum (biological and technical replicates). **A**: All strains from IAL and FMVZ-USP classified by serovars; **B**: All strains from IAL and FMVZ-USP classified by serogroups

Figure 2 shows the overall PCA analysis including all tested strains (reference strains maintained at IAL and FMVZ-USP). Panel A illustrates clustering at the serovar level, whereas Panel B reflects broader relationships at the serogroup level. The results of PCA analysis revealed clustering patterns. When the data were grouped by serovar (Fig. 2A), distinct clusters were observed, with partial overlap among some serovars. Similarly, grouping by serogroup (Fig. 2B) resulted in cluster formation, with separation among serogroups and partial overlap between certain groups.

Replicate consistency was also evaluated across serovars by considering both technical replicates (five spectra obtained per run) and biological replicates (three independent experiments performed from successive weekly subcultures). Among the tested groups, strains belonging to the Sejroe serogroup showed the highest spectral reproducibility. Both technical and biological replicates formed compact and well-defined clusters (Fig. 1C). In contrast, serovars from the Icterohaemorrhagiae and Australis serogroups exhibited greater dispersion among replicates.

## Discussion

This study reports the first application of Fourier-transform infrared (FT-IR) spectroscopy using the IR Biotyper^®^ system for the characterization of *Leptospira* strains. FT-IR generates spectral fingerprints based on molecular vibrations that reflect the biochemical composition of cellular constituents, including lipopolysaccharides (LPS), which are traditionally used for *Leptospira* serovar differentiation [3, 14, 15]. The IR Biotyper^®^ offers a standardized, rapid, and reproducible workflow, features that are advantageous for routine laboratory implementation. Previous studies applying this technology to organisms such as *Klebsiella pneumoniae*, *Pseudomonas aeruginosa*, and others have demonstrated its potential as a rapid typing tool with discriminatory capacity that in some cases parallels conventional genomic and molecular methods, although performance can vary depending on the organism and analytical approach [24–28]. Rapid and reliable strain characterization is particularly relevant for leptospirosis surveillance, where serovar identification remains essential for epidemiological investigations.

Whole-genome sequencing (WGS) was employed in this study as a reference method to confirm serovar identification, thereby supporting the validation of the FT-IR-based classification and its discriminatory capacity. Multiple taxonomic tools were evaluated following WGS, providing a robust framework for comparison. While all methods achieved reliable species-level classification, differences in serovar-level resolution were evident. Notably, the jSpecies platform, based on average nucleotide identity (ANI) Z-score analysis, was able to distinguish closely related serovars such as Icterohaemorrhagiae and Copenhageni, which often share the same sequence type (ST) by MLST [16]. This observation aligns with previous genomic work identifying distinct clusters and a phase-shift mutation in the *lic12008* gene in Icterohaemorrhagiae [22].

Despite the fastidious growth requirements of *Leptospira* spp., the optimized sample preparation protocol produced spectra suitable for analysis while ensuring biosafety through chemical inactivation. Of the 330 spectra generated, most (73.03%) achieved Green quality scores, while 26.96% were classified as Orange, predominantly due to a problem of absorbance associated with insufficient biomass (74 spectra), with fewer cases related to homogenization issues (six spectra) or both factors (nine spectra). These findings likely reflect the known variability in growth kinetics and biomass yield among *Leptospira* strains, indicating that adjustments in inoculum volume and culture density may be necessary when applying this protocol to different strains or laboratory settings.

PCA demonstrated discrimination of serovars within individual serogroups, whereas the combined analysis including all strains revealed partial overlap among certain serovars and serogroups. This pattern is biologically plausible and likely reflects the structural relatedness among closely related *Leptospira* serovars. Serological classification in *Leptospira* is primarily determined by variations in lipopolysaccharide (LPS) structure, and substantial antigenic homology exists between serovars belonging to the same serogroup. Such structural similarity likely contributes to the observed overlap and is consistent with the well-documented cross-reactivity of anti-Leptospira antibodies in serological assays, particularly in the microscopic agglutination test (MAT) [3, 7].

Despite this structural similarity, subtle biochemical differences may still allow discrimination between closely related serovars. In this context, recent work by Lewicka et al. (2025) [15] demonstrated that variations in LPS saccharide composition can enable differentiation of antigenically similar *Leptospira* serovars. These findings provide a biochemical basis that supports the spectral differentiation observed using FT-IR in the present study, even among closely related serovars.

An additional observation was the greater spectral reproducibility for strains belonging to the Sejroe serogroup, where both technical and biological replicates consistently formed compact clusters. In contrast, greater dispersion of replicates was observed for serovars of the Icterohaemorrhagiae and Australis serogroups. Although the reasons underlying this pattern remain unclear, it may reflect differences in phenotypic stability or molecular surface composition among serogroups [7]. Further studies including a larger number of strains will be necessary to determine whether this observation represents a biological characteristic of these serogroups or reflects variability among individual strains. Importantly, all strains were cultivated under identical growth conditions, suggesting that the observed spectral differences are unlikely to be attributable to variations in culture parameters.

Some limitations should be acknowledged. This study represents a preliminary protocol development investigation with a limited number of strains, including only one representative strain per serovar. Therefore, the clustering observed likely reflects differences between individual isolates rather than the full variability within each serovar, and does not allow assessment of intra-serovar variability or confirmation of reproducible serovar discrimination across multiple independent isolates. In addition, although PCA provided useful exploratory insight into spectral patterns, larger strain collections and supervised classification models will be required to more rigorously evaluate the robustness and predictive performance of this approach.

Despite these limitations, this preliminary protocol development study demonstrates that FT-IR spectroscopy using the IR Biotyper^®^ system represents a feasible, rapid, and reproducible approach for the characterization of *Leptospira* strains. The optimized workflow ensured biosafety and generated consistent spectral data, providing preliminary evidence that FT-IR may have potential for differentiating epidemiologically relevant serovars, including closely related serovars such as Icterohaemorrhagiae and Copenhageni.

## Conclusions

This study demonstrates the feasibility of applying FT-IR spectroscopy using the IR Biotyper^®^ system for the characterization of *Leptospira* spp. Following optimization of a sample preparation protocol adapted to liquid EMJH cultures, reproducible spectra were obtained from multiple serovars representing epidemiologically relevant serogroups, with clustering patterns suggesting potential for differentiation of closely related serovars. Although preliminary, these findings indicate that FT-IR may represent a promising rapid phenotypic approach for *Leptospira* characterization, warranting further validation using larger and more diverse strain collections.

## Supplementary Information

Below is the link to the electronic supplementary material.

Supplementary Material 1.

Supplementary Material 2.

Supplementary Material 3.

Supplementary Material 4.

Supplementary Material 5.

## Data Availability

The genomic data generated in this study have been deposited in the NCBI database under BioProject accession number PRJNA1423741. FT-IR spectra and derived PCA datasets generated during the current study are available from the corresponding author upon reasonable request.

## References

[1] Haake DA, Levett PN (2015) Leptospirosis in humans. Curr Top Microbiol Immunol 387:65–97. 10.1007/978-3-662-45059-8_5 25388133 10.1007/978-3-662-45059-8_5 PMC4442676

[2] Polo N, Machado G, Rodrigues R, Nájera Hamrick P, Munoz-Zanzi C, Pereira MM, Bercini M, Timm LN, Schneider MC (2019) A One Health Approach to Investigating Leptospira Serogroups and Their Spatial Distributions among Humans and Animals in Rio Grande do Sul, Brazil, 2013–2015. Trop Med Infect Dis 4(1):42. 10.3390/tropicalmed4010042 30818803 10.3390/tropicalmed4010042 PMC6473481

[3] Faine S, Adler B, Bolin C, Perolat P (1999) Leptospira and Leptospirosis. 2nd Edition, Medisci Press, Melbourne

[4] Ko AI, Galvão RM, Ribeiro DCM, Johnson WD, Jr, Riley LW (1999) Urban epidemic of severe leptospirosis in Brazil. Lancet 354(9192):1735–1737. 10.1016/s0140-6736(99)80012-9 10.1016/s0140-6736(99)80012-9 10485724

[5] Romero EC, Bernardo CC, Yasuda PH (2003) Human leptospirosis: a twenty-nine-year serological study in São Paulo, Brazil. Rev Inst Med Trop São Paulo 45(5):245–248. 10.1590/s0036-46652003000500002 14743663 10.1590/s0036-46652003000500002

[6] Bradley EA, Lockaby G (2023) Leptospirosis and the Environment: A Review and Future Directions. Pathogens 12(9):1167. 10.3390/pathogens12091167 37764975 10.3390/pathogens12091167 PMC10538202

[7] Levett PN (2001) Leptospirosis. Clin Microbiol Ver 14(2):296–326. 10.1128/CMR.14.2.296-326 10.1128/CMR.14.2.296-326.2001 PMC88975 11292640

[8] Sonada RB, Azevedo SS, Soto FRM, Costa DFD, Morais ZM, Souza GO, Gonçales AP, Miraglia F, Vasconcellos SA (2018) Efficacy of leptospiral commercial vaccines on the protection against an autochtonous strain recovered in Brazil. Braz J Microbiol 49(2):347–350. 10.1016/j.bjm.2017.06.008 29122476 10.1016/j.bjm.2017.06.008 PMC5913823

[9] Barazzone GC, Teixeira AF, Azevedo BOP, Damiano DK, Oliveira MP, Nascimento ALTO, Lopes APY (2022) Revisiting the Development of Vaccines Against Pathogenic Leptospira: Innovative Approaches, Present Challenges, and Future Perspectives. Front Immunol 3(12):760291. 10.3389/fimmu.2021.760291 10.3389/fimmu.2021.760291 PMC8761801 35046936

[10] Bergmann ES, Moreira SC, Ferreira SF, Gonçales AP, Guilloux AGA, Marinelli CM, Hagiwara MK, Miotto BA (2022) Efficacy of commercially available vaccines against canine leptospirosis: A systematic review and meta-analysis. Vaccine 40(12):1722–1740. 10.1016/j.vaccine.2022.02.021 35153090 10.1016/j.vaccine.2022.02.021

[11] Goris MG, Hartskeerl RA (2014) Leptospirosis serodiagnosis by the microscopic agglutination test. Curr Protoc Microbiol 32(Unit 12E5). 10.1002/9780471729259.mc12e05s32 10.1002/9780471729259.mc12e05s32 24510846

[12] Pyskun O, Richter MH (2025) Look and you will find-a literature review of new strains of Leptospira spp., 2000–2025. FEMS microbiology reviews, 49, fuaf054. 10.1093/femsre/fuaf054 10.1093/femsre/fuaf054 PMC12629226 41206557

[13] Arent Z, Gilmore C, Pardyak L, Dubniewicz K, McInerney B, Ellis W (2023) The serological and genetic diversity of the Leptospira interrogans Icterohaemorrhagiae serogroup circulating in the UK. J Vet Res 67(4):529–536. 10.2478/jvetres-2023-0063 38130449 10.2478/jvetres-2023-0063 PMC10730551

[14] Vanithamani S, Mercy A, Kanagavel CS, Sumaiya M, Bothammal K, Saranya P, Prasad P, Ponmurugan M, Muralitharan K, Al-Dhabi G, Verma NA, Vijayachari A, Natarajaseenivasan P, K (2021) Biochemical analysis of leptospiral LPS explained the difference between pathogenic and non-pathogenic serogroups. Microb Pathog 152:104738. 10.1016/j.micpath.2021.104738 33529737 10.1016/j.micpath.2021.104738

[15] Lewicka AJ, Lyczakowski JJ, Pardyak L, Dubniewicz K, Latowski D, Arent Z (2025) Beyond serology: saccharide profiling enables identification of antigenically similar Leptospira and prompts re-evaluation of bacterial lipopolysaccharide evolution. Front Mol Biosci 12:1581587. 10.3389/fmolb.2025.1581587 40600027 10.3389/fmolb.2025.1581587 PMC12208846

[16] Romero EC, Blanco RM, Galloway RL (2011) Analysis of multilocus sequence typing for identification of Leptospira isolates in Brazil. J Clinl Microbiol 49(11):3940–3942. 10.1128/JCM.01119-11 10.1128/JCM.01119-11 PMC3209071 21880969

[17] Sykes JE, Francey T, Schuller S, Stoddard RA, Cowgill LD, Moore GE (2023) Updated ACVIM consensus statement on leptospirosis in dogs. J Vet Intern Med 37(6):1966–1982. 10.1111/jvim.16903 37861061 10.1111/jvim.16903 PMC10658540

[18] Browne ES, Callefe JLR, Jesus ERS, Zeppelini CG, Cremonese C, Costa FA (2022) A Systematic Review of the geographic distribution of pathogenic Leptospira serovars in the Americas, 1930–2017. Acad Bras Ciênc 94(3):e20201026. 10.1590/0001-3765202220201026 10.1590/0001-3765202220201026 36074401

[19] Jaeger LH, Pestana CP, Correia LFL, Carvalho-Costa FA, Medeiros MA, Lilenbaum W (2019) Novel MLST sequence types of pathogenic Leptospira spp.: Opening the black box of animal leptospirosis in Brazil. Acta Trop 196:135–141. 10.1016/j.actatropica.2019.05.025 31121146 10.1016/j.actatropica.2019.05.025

[20] Machry RB et al (2010) Characterization of Leptospira sp reference strains using the pulsed field gel electrophoresis technique. Rev Soc Bras Med Trop 43(2):166–169. 10.1590/s0037-86822010000200012 20464147 10.1590/s0037-86822010000200012

[21] Romero EC, Blanco RM, Galloway RL (2009) Application of pulsed-field gel electrophoresis for the discrimination of leptospiral isolates in Brazil. Lett Appl Microbiol 48(5):623–627. 10.1111/j.1472-765X.2009.02580.x 19416464 10.1111/j.1472-765X.2009.02580.x

[22] Santos LA, Adhikarla H, Yan X, Wang Z, Fouts DE, Vinetz JM, Alcantara LCJ, Hartskeerl RA, Gori MGA, Picardeau M, Reis MG, Townsend JP, Zhao H, Ko AI, Wunder EA Jr (2018) Genomic Comparison Among Global Isolates of L. interrogans Serovars Copenhageni and Icterohaemorrhagiae Identified Natural Genetic Variation Caused by an Indel. Front Cell Infect Microbiol 19(8):193. 10.3389/fcimb.2018.00193 10.3389/fcimb.2018.00193 PMC6018220 29971217

[23] Muchaamba F, Stephan R (2024) A Comprehensive Methodology for Microbial Strain Typing Using Fourier-Transform Infrared Spectroscopy. Methods Protoc 7(3):48. 10.3390/mps7030048 38921827 10.3390/mps7030048 PMC11207048

[24] Burckhardt I, Sebastian K, Mauder N, Kostrzewa M, Burckhardt F, Zimmermann S (2019) Analysis of Streptococcus pneumoniae using Fourier-transformed infrared spectroscopy allows prediction of capsular serotype. Eur J Clin Microbiol Infect Dis 38(10):1883–1890. 10.1007/s10096-019-03622-y 31286288 10.1007/s10096-019-03622-y PMC6778537

[25] Martak D, Valot B, Sauget M, Cholley P, Thouverez M, Bertrand X, Hocquet D (2019) Fourier-Transform InfraRed Spectroscopy Can Quickly Type Gram-Negative Bacilli Responsible for Hospital Outbreaks. Front Microbiol 10:1440. 10.3389/fmicb.2019.01440 31293559 10.3389/fmicb.2019.01440 PMC6606786

[26] Park S, Ryoo N (2024) Comparative analysis of IR-Biotyper, MLST, cgMLST, and WGS for clustering of vancomycin-resistant Enterococcus faecium in a neonatal intensive care unit. Microbiol Spectr 12(e04119–23). 10.1128/spectrum.04119-23 10.1128/spectrum.04119-23 PMC10986520 38441473

[27] Pascale MR, Bisognin F, Mazzotta M, Girolamini L, Marino F, Monte D, Cordovana P, Scaturro M, Ricci M, Cristino ML, S (2022) Fourier-transform infrared spectroscopy (FT-IR) for typing Legionella pneumophila serogroups: a rapid and high-throughput alternative. Front Microbiol 11(1):1993–2003. 10.3389/fmicb.2022.866426 10.3389/fmicb.2022.866426 PMC9090449 35558114

[28] Rakovitsky N, Frenk S, Kon H, Schwartz D, Temkin E, Solter E, Paikin S, Cohen R, Schwaber MJ, Carmeli Y, Lellouche J (2020) Fourier Transform Infrared Spectroscopy Is a New Option for Outbreak Investigation: A Retrospective Analysis of an Extended-Spectrum-Beta-Lactamase-Producing Klebsiella pneumoniae Outbreak in a Neonatal Intensive Care Unit. J Clin Microbiol 58(5):e00098–e00020. 10.1128/jcm.00098-20 32161093 10.1128/JCM.00098-20 PMC7180251

[29] Richter M, Rosselló Móra R (2009) Shifting the genomic gold standard for the prokaryotic species definition. Proc Natl Acad Sci U S A 106(45):19126–19131. 10.1073/pnas.0906412106 19855009 10.1073/pnas.0906412106 PMC2776425

[30] Clausen PTLC, Aarestrup FM, Lund O (2018) Rapid and precise alignment of raw reads against redundant databases with KMA. BMC Bioinformatics 19(1):307. 10.1186/s12859-018-2336-6 30157759 10.1186/s12859-018-2336-6 PMC6116485

[31] Argimón S, David S, Underwood A, Abrudan M, Wheeler NE, Kekre M, Abudahab K, Yeats CA, Goater R, Taylor B, Harste H, Muddyman D, Feil EJ, Brisse S, Holt K, Donado-Godoy P, Ravikumar KL, Okeke IN, Carlos C, Aanensen DM, NIHR Global Health Research Unit on Genomic Surveillance of Antimicrobial Resistance (2021) Rapid Genomic Characterization and Global Surveillance of Klebsiella Using Pathogenwatch. Clin Infect Dis 73(4):325–335. 10.1093/cid/ciab784 10.1093/cid/ciab784 PMC8634497 34850838

[32] Pope V, Johnson RC (1987) Effect of heat or chemical treatment on leptospiral antigens. J Clin Microbiol 25(2):255–258. 10.1128/jcm.25.2.255-258.1987 3818922 10.1128/jcm.25.2.255-258.1987 PMC265878

