# Supplementary material for: Application of FT-IR spectroscopy using the IR Biotyper® for Leptospira: protocol optimization and first spectral insights
Source: Eur J Clin Microbiol Infect Dis. 2026 Apr 11;45(8):2281–90. doi: 10.1007/s10096-026-05507-3 (PMC13428689; doi:10.1007/s10096-026-05507-3)
Supplement: Supplementary file 2 — Supplementary Material 2. [file 10096_2026_5507_MOESM2_ESM.docx]

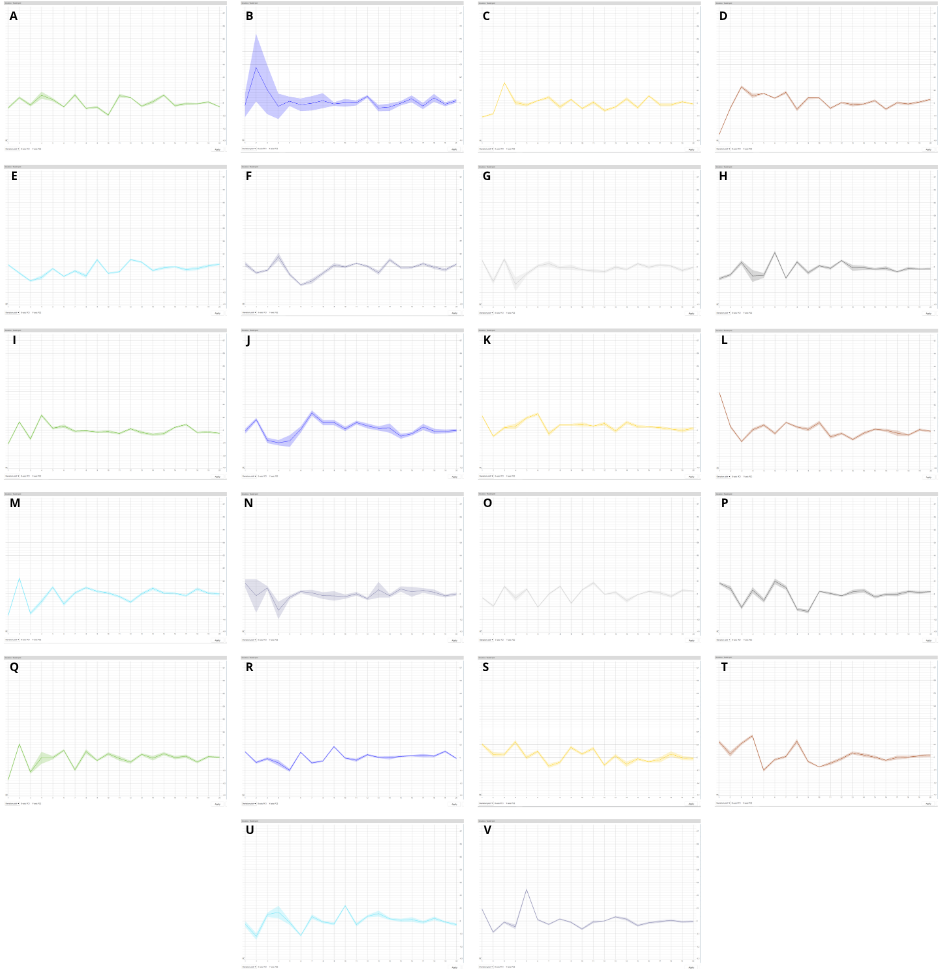


**Supplementary Figure 1.** Representative FT-IR spectra of Leptospira serovars analyzed in this study.

Each panel (A–V) displays a typical spectrum obtained for one representative strain of each serovar included in the analysis. Spectra correspond to the carbohydrate fingerprint region (1300–800 cm⁻¹) and are shown as raw, non-averaged profiles to illustrate spectral variability across replicates.

The correspondence between panels and serovars is as follows:

**A:** Australis**; B:** Autumnalis; **C:** Bataviae; **D:** Bratislava; **E:** Canicola; **F:** Castellonis; **G:** Copenhageni; **H:** Cynopteri; **I:** Djasiman; **J:** Grippotyphosa; **K:** Hardjo; **L:** Hardjobovis; **M:** Hebdomadis; **N:** Icterohaemorrhagiae; **O:** Javanica; **P:** Panama; **Q:** Pomona; **R:** Pyrogenes; **S:** Sejroe; **T:** Tarassovi; **U:** Whitcombi; **V:** Wolffi.
