# Supplementary figures and images for: Application of FT-IR spectroscopy using the IR Biotyper® for Leptospira: protocol optimization and first spectral insights
Source: Eur J Clin Microbiol Infect Dis. 2026 Apr 11;45(8):2281–90. doi: 10.1007/s10096-026-05507-3 (PMC13428689; doi:10.1007/s10096-026-05507-3)

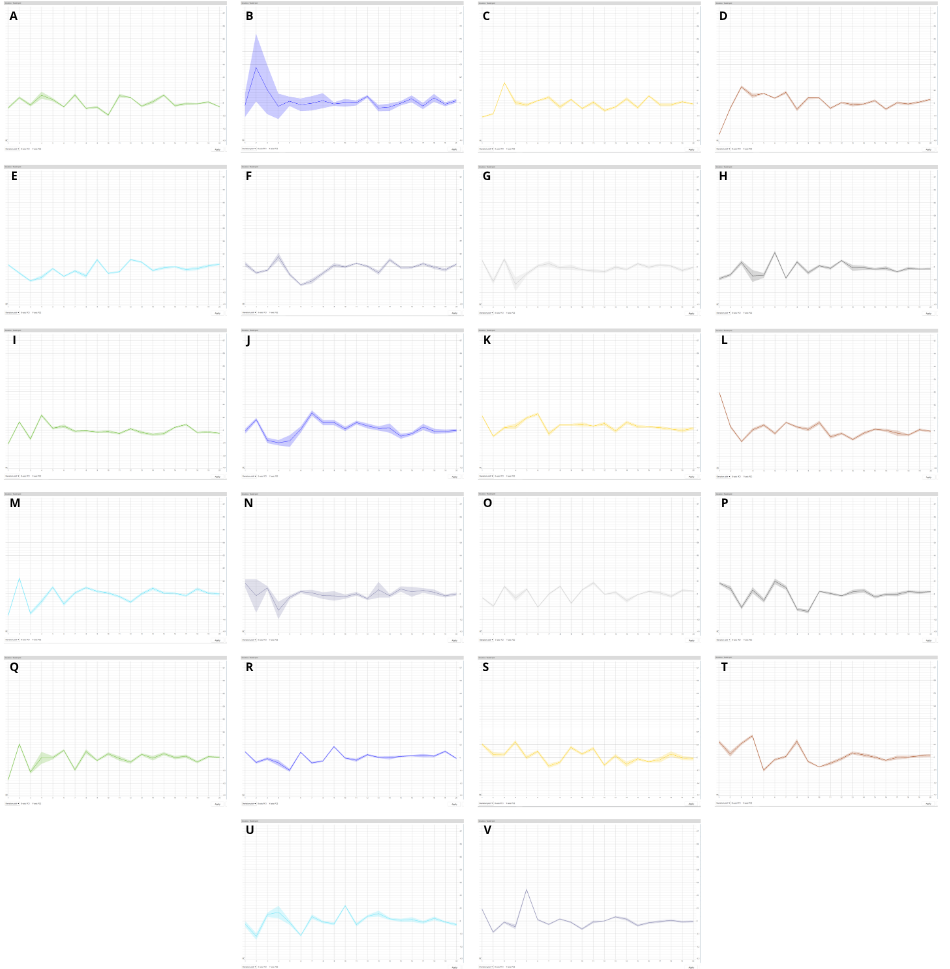

Supplement: Supplementary file 3 — Supplementary Material 3. [file 10096_2026_5507_MOESM3_ESM.tif]

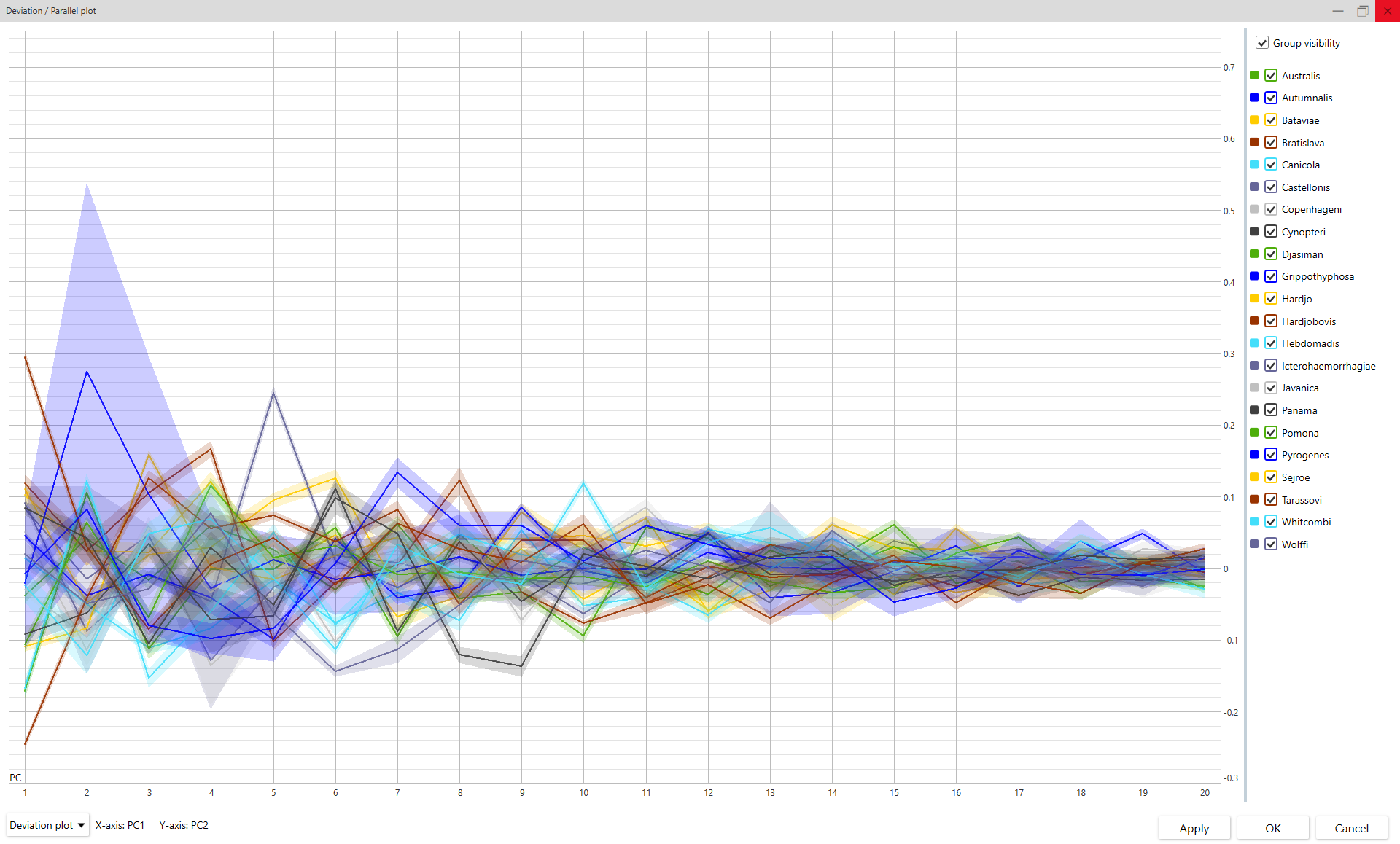

Supplement: Supplementary file 5 — Supplementary Material 5. [file 10096_2026_5507_MOESM5_ESM.tif]
