# Supplementary material for: Application of FT-IR spectroscopy using the IR Biotyper® for Leptospira: protocol optimization and first spectral insights
Source: Eur J Clin Microbiol Infect Dis. 2026 Apr 11;45(8):2281–90. doi: 10.1007/s10096-026-05507-3 (PMC13428689; doi:10.1007/s10096-026-05507-3)
Supplement: Supplementary file 4 — Supplementary Material 4. [file 10096_2026_5507_MOESM4_ESM.docx]

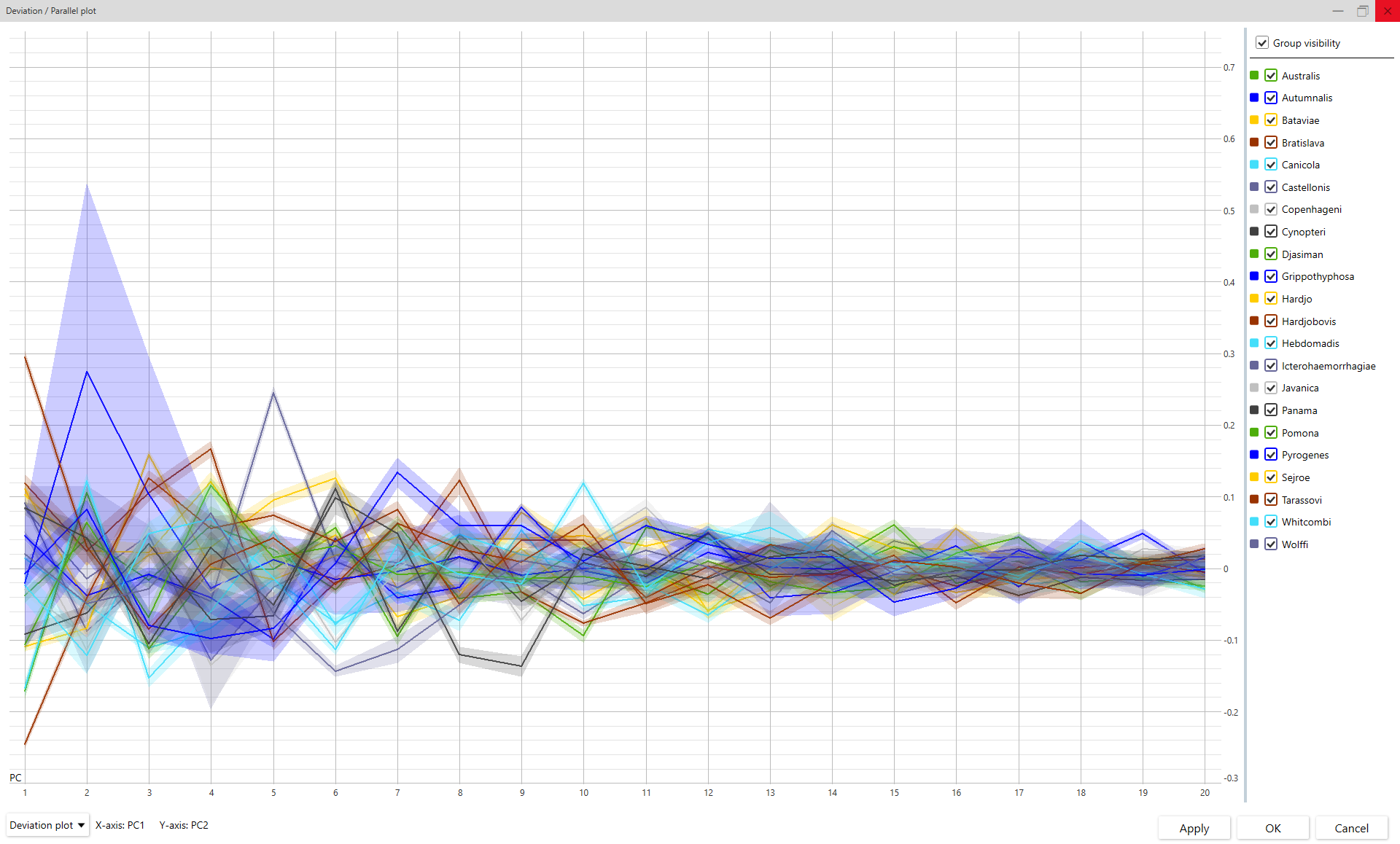


**Supplementary Figure 2.** Comparative FT-IR spectra of all Leptospira serovars included in the study. This figure displays the representative FT-IR spectra of all analyzed serovars overlaid in a single plot to allow direct visual comparison of their spectral profiles. Spectra correspond to the carbohydrate fingerprint region (1300–800 cm⁻¹) and are shown as typical profiles derived from the optimized experimental conditions. Differences in peak intensity and distribution highlight the spectral variability among serovars.
